# Dynamical crises, multistability and the influence of the duration of immunity in a seasonally-forced model of disease transmission

**DOI:** 10.1186/1742-4682-11-43

**Published:** 2014-10-04

**Authors:** Mathew P Dafilis, Federico Frascoli, Jodie McVernon, Jane M Heffernan, James M McCaw

**Affiliations:** Centre for Epidemiology and Biostatistics, Melbourne School of Population and Global Health, The University of Melbourne, Melbourne, VIC Australia; Murdoch Childrens Research Institute, Royal Children’s Hospital, Parkville, VIC Australia; Department of Mathematics, Swinburne University of Technology, Hawthorn, VIC Australia; Modelling Infection and Immunity Lab, Centre for Disease Modelling, York Institute for Health Research, York University, Toronto, ON Canada; Mathematics & Statistics, York University, Toronto, ON Canada

**Keywords:** Infectious diseases, Mathematical model, Immunity, Dynamical crises

## Abstract

**Background:**

Highly successful strategies to make populations more resilient to infectious diseases, such as childhood vaccinations programs, may nonetheless lead to unpredictable outcomes due to the interplay between seasonal variations in transmission and a population’s immune status.

**Methods:**

Motivated by the study of diseases such as pertussis we introduce a seasonally-forced susceptible-infectious-recovered model of disease transmission with waning and boosting of immunity. We study the system’s dynamical properties using a combination of numerical simulations and bifurcation techniques, paying particular attention to the properties of the initial condition space.

**Results:**

We find that highly unpredictable behaviour can be triggered by changes in biologically relevant model parameters such as the duration of immunity. In the particular system we analyse — previously used in the literature to study pertussis dynamics — we identify the presence of an initial-condition landscape containing three coexisting attractors. The system’s response to interventions which perturb population immunity (e.g. vaccination "catch-up" campaigns) is therefore difficult to predict.

**Conclusion:**

Given the increasing use of models to inform policy decisions regarding vaccine introduction and scheduling and infectious diseases intervention policy more generally, our findings highlight the importance of thoroughly investigating the dynamical properties of those models to identify key areas of uncertainty. Our findings suggest that the often stated tension between capturing biological complexity and utilising mathematically simple models is perhaps more nuanced than generally suggested. Simple dynamical models, particularly those which include forcing terms, can give rise to incredibly complex behaviour.

## Introduction

Simple dynamical models of infectious disease have helped explain the spectacular success of childhood immunisation programs, yielded valuable insights into complex epidemiological phenomena [1–3] and contributed to public health policy development [4–10]. In particular, Earn *et al.* demonstrated that transitions from predictable to unpredictable, and potentially chaotic, behaviour for childhood diseases such as measles may arise naturally from changes in birth rates and/or vaccination schedules [3]; while Lavine *et al.* demonstrated that under a hypothesis whereby immunity to pertussis is maintained through regular exposure to infectious individuals, childhood vaccination may induce, rather than suppress, periodic oscillations in the incidence of disease [11], and possibly result in co-existing dynamical regimes (multistability). Recently, we have examined the behaviour of the model used by Lavine *et al.* and demonstrated that similarly complex patterns of infection may arise even in the absence of vaccination. In particular, we demonstrated that changes in the birth-rate, as naturally occurred with the 20th Century demographic transition in Western populations, may have transitioned the dynamical system from one in which endemic steady-state dynamics existed to one in which sustained (undamped) oscillations and multistability may characterise the system [12].

Our study [12] as well as those by Earn and Lavine indicate that the *predicted* broad-brush characteristic behaviours of infectious diseases in the human population depend critically upon the assumed mechanism(s) of i) infection; ii) the development and maintenance of immunity; iii) the (changing) demographic state of the population; and iv) the particular form of the equations used to model the underlying dynamical system.

It is well understood that the infectiousness of many diseases varies over the course of the year — as a result of numerous factors including the effects of temperature and humidity on the survival of the pathogen and the influence of weather on human behaviour [13–15]. It follows that for many infectious disease studies it is appropriate to include a ‘seasonal-forcing’ term that periodically modulates the intensity of the transmission process [3, 16, 17]. It is also well known that subjecting a dynamical system to an external driving force has the potential to induce complex and likely chaotic behaviour in what were previously ‘well-behaved’ predictable dynamical systems [17–19]. In a recent study [20] we examined the complicated dynamical behaviour arising from a seasonally-forced extension to the model [11, 12] previously used to consider pertussis dynamics. We characterised the dynamics of infection as a function of demographic parameters (birth and death rates), the strength of immune boosting and the strength of seasonal-forcing. We described how forcing induces sustained periodic oscillations and identified period-doubling routes to chaos. We related the findings back to dynamical properties of the unforced-system.

Here we explore a particular aspect of this model’s behaviour in greater detail, identifying so-called dynamical ‘crisis’, characterised by infinitely complicated basins of attraction, outbursts and aperiodic oscillatory behaviour. Our main finding is that variations in parameters of biological significance — illustrated here through systematic variation in the duration of immunity — are responsible for sudden and unpredictable changes in the model dynamics, making the use of such models as *predictive tools* fraught with difficulty. Our findings build upon earlier studies of the properties of measles transmission models by Ferguson *et al.* [21] and Aron [22].

## Methods

### The SIRWS model

**Figure 1:**
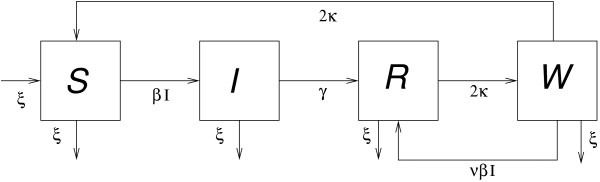
**The SIRWS model.**

Extending the standard epidemic theory — and as introduced by Lavine in the context of pertussis — the model consists of four population compartments: susceptible (*S*), infectious (*I*), recovered (*R*) and a waning immunity state (*W*). Those in the waning state (*W*) can either lose their immunity and return to *S* (hence the model name: SIRWS), or alternatively have their immunity boosted at some rate *νβI* (proportional to the force of infection), and return to *R*. Seasonality is incorporated into the model by cyclically modulating the force of infection. Figure 1 presents a schematic of the model. The non-linear differential equations governing the system are given by:
1a1b1c1d

where *β*(*t*) = *β*_0_(1 + *η* cos(2*πt*)) is the annually-forced transmission coefficient, parameterised by a baseline value *β*_0_ and a seasonal strength *η*, 1/*γ* is the average duration of infectiousness, 1/*κ* is the average duration of protection (in the absence of immune boosting), *ν* is the factor describing the relative strength of immune boosting (*W* → *R*) compared to infection (*S* → *I*) and *ξ* is the birth rate for the population.

Critically, following both Lavine *et al.* [11] and Dafilis *et al.* [12], we allow for *ν* > 1 with the implication that individuals with intermediate levels of immunity have a heightened sensitivity to boosting when exposed to infection compared with *susceptible* individuals. i. e., an exposure insufficient to initiate productive infection in a susceptible individual may nonetheless be sufficient to boost an individual’s immunity, returning them from the *W* to *R* state. The presence of this extra non-linear feedback loop in the system has fundamental consequences for its dynamics, as previously explored in detail by the present authors [12].

Here, our focus will be on how the duration of immune protection, one of the most poorly understood aspects of pertussis epidemiology, influences the dynamics of the system and how biologically reasonable choices for that duration can lead to intricate and hard-to-characterise basins of attraction for the model. To undertake the study we must choose a set of biologically realistic parameters with which to simulate the model. In other work [20] we have explored the dynamics of the system over parameter-space, considering how the amplitude of seasonal-forcing and strength of immune boosting influence the dynamics. Based on those extensive studies, we have chosen a single representative set of parameters (other than the duration of immunity which we vary here) which are compatible with the current literature and observed pertussis epidemiology [11, 12, 23] and yet support chaotic infection dynamics [20]. The infectious duration (in the absence of births and deaths) is set to 1/*γ* = 1/17 years (i.e. 21 days). Baseline infectiousness is set to *β*_0_ = 260 years^-1^ yielding a basic reproduction number of 15.2. The strength of seasonal-forcing is set to 16% (*η* = 0.16). The birth rate is set to 1/100 years^-1^, corresponding to an average lifespan of 100 years. Based on a recent analysis of pertussis in Copenhagen by Lavine *et al.* [23] we set immune boosting to be more likely to occur than infection (*ν* = 5). Throughout the paper, the duration of immunity is either fixed at 10 years (*κ* = 1/10 years^-1^) or varied over the range [1,20] years.

### Analysis

**Figure 2:**
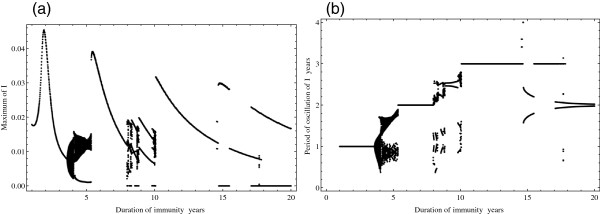
**Stability, dynamical crises and chaos characterise the SIRWS system as the duration of immunity is varied between 1 and 20 years.** The peak prevalence **(a)** and the period of oscillation **(b)**, each plotted as a function of the duration of immunity (1/*κ*) are shown. Crises, quasiperiodicity, and chaos are all evident as the duration of immunity is varied. Note the sudden jump in the maximum of *I* nearby a duration of immunity of 10 years. This value happens to be that previously chosen in the literature [11, 12] to model pertussis dynamics indicating the presence of a crisis nearby an epidemiologically favoured value for the duration of immunity. The figures were produced by integrating the system for sufficient time such that the transient dynamics had passed (*t*_*transient*_ = 1000yr) and then examining the time traces over the next 50 years of simulation. The peak prevalence was calculated as the maximum of the oscillation of *I* and the period of oscillation as the time between successive maxima of *I*.

**Figure 3:**
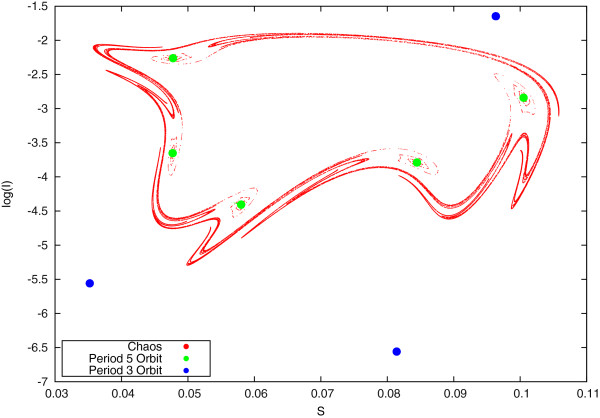
**A stroboscopic plot of the three attractors in a** ***S*** **– log** ***I*** **phase space cross section.** The duration of immunity is set at 10 years, and all other parameters are as specified in the text. Different colours indicate different attractors, with red showing the chaotic attractor, green a period 5 orbit and blue a period 3 orbit.

**Figure 4:**
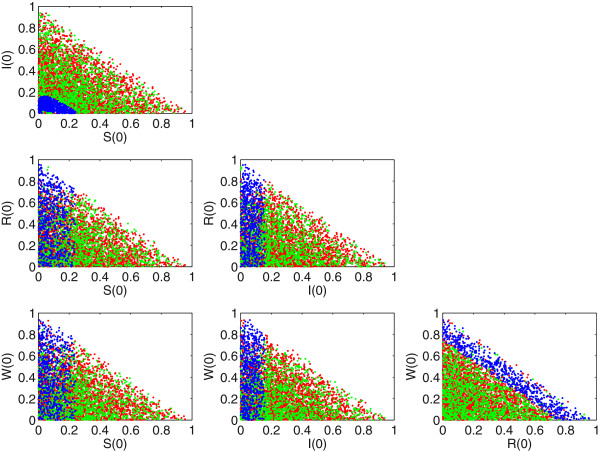
**Two-dimensional slices through the full four-dimensional initial-condition space.** Parameter values are fixed as per Figure 3. Period-3 attractors are labelled in blue, period-5 attractors in green and chaotic attractors in red. White is empty space where there was no sample taken. The initial-condition space is restricted to the lower triangular region due to the condition *S*(0) + *I*(0) + *R*(0) + *W*(0) = 1. 5000 randomly selected data points are presented from the complete 250,000 simulations performed for clarity of presentation. The relative proportions of each type of attractor determined from the full data set were preserved.

The system of coupled differential equations 1 was solved using the ODE MEX interface by Vanlier [24]. Seasonality was incorporated as in [16], allowing for a completely autonomous oscillator, which aided the analysis. Integration time was 2000 years, with the first 1000 years discarded as a transient. The period of the oscillation of the infectious state (*I*) time series was determined using the period detection algorithm of Taylor [25] applied to a 50 year time interval. As discussed in detail in [20], for multi-year periodic cycles there may be multiple local maxima within the cycle. We define the peak prevalence as the maximum value obtained over the entire cycle. Note that in previous work [20] we examined the properties of these multi-year periodic cycles in detail. Where the period detection algorithm failed to give a determination of the period, the largest Lyapunov exponent of the system for the particular set of initial conditions and parameters was evaluated using the algorithm of Christiansen [26] applied to the last 1000 years of the simulation. The presence of chaos was also confirmed by an application of the Gottwald-Melbourne 0–1 test for both *S* and *I* time series [27–31]. Model parameters were fixed to *β*_0_ = 260 years^-1^, *γ* = 17 years^-1^, *κ* = 1/10 years^-1^, *ξ* = 1/100 years^-1^ and *ν* = 5 unless specified otherwise, in line with previous work [11, 12] and broadly consistent with a model of pertussis infection. The strength of seasonal-forcing was fixed at *η* = 0.16, which is a chaotic point in parameter space. The period of forcing was always set to one year. The initial conditions for the simulations presented in Figures 2(a) and 2(b) were fixed to a fully susceptible population (*S*(0) = 0.99, *I*(0) = 0.01, *R*(0) = 0, *W*(0) = 0). Data for Figures 2(a) and 2(b) were obtained using XPPAUT [32], with post-processing in *Mathematica*. Data for Figure 3 were drawn from the last 1000 years of the simulation and prepared using *Mathematica*. In Figure 4 the parameter set was fixed and the initial conditions selected uniformly at random, consistent with *S*(0) + *I*(0) + *R*(0) + *W*(0) = 1.

## Results

### Identifying dynamical crises: The influence of the duration of immunity on steady-state dynamics

The sensitivity of the system to biologically realistic variation in the duration of immunity (1/*κ*) is shown through a quasi-bifurcation diagram (Figure 2(a)) for the value of the peak prevalence of infection, *I*, obtained over a 50 year time period in which the system is in a dynamic steady-state. Figure 2(b) shows the corresponding plot for the period of the oscillations of the 50 year period. Focussing first on Figure 2(a), as the duration of immunity is increased from 1 year the value of the peak prevalence varies smoothly with the duration of immunity up until approximately 3.5 years, after which a clustering of many maxima is noticed. This observation is indicative of an aperiodic oscillation over the 50 year period under study, consistent with our initial numerical findings [20]. Notably, in the proximal neighbourhood of 1/*κ*=5.5 years, a large transition in the peak prevalence is evident: this is the outbreak of a so-called ‘crisis’ [21, 33, 34], which signals a dramatic and sudden change in the nature of the dynamics (i.e. in the character of the corresponding attractor). The return to a smooth variation in the maxima is similarly sudden. After a brief, smooth increase in the peak prevalence, a continuous decrease is observed as 1/*κ* increases from approximately 6 years towards 8 years, indicating a range of values for the duration of protection for which periodic oscillations are sustained over the 50 year period under study. As the duration of immunity is increased beyond 8 years, the system again presents narrow intervals for 1/*κ* for which the dynamics are unpredictable and multiple ‘crises’ are evident. The dynamical features just described are similarly evident in the plot of the period of oscillations (Figure 2(b)), where the influence of the seasonal-forcing is evident. For a duration of immunity less than 3.5 years, the system displays annual oscillations (Figure 2(b)). It then enters a regime in which the dynamics are aperiodic before jumping, at 1/*κ* approximately equal to 5.5 years, to bi-annual (period 2) outbreaks. Separated by regions of complex behaviour, for durations of immunity between 10 and 15 years, the system supports tri-annual (period 3) outbreaks. Mathematically, the sudden, unpredictably and irregular transitions are a consequence of the highly non-linear character of the model and the way it responds to external forcing, as previously discussed in [20].

It is well established that close to a crisis (such as those present in our model) complicated basins of attraction may often be found [21, 22, 34]. In consequence, the initial choice of how to populate the susceptible (*S*), infectious (*I*), recovered (*R*) and waned-immunity (*W*) states in our model may deeply influence the nature of the long term behaviour of the model. We now explore these possibilities in detail.

### Riddled basins: the complex dependence on initial conditions at a crisis point

We fix the duration of immunity (1/*κ*) to 10 years — in accordance with Lavine *et al.*’s study of pertussis [11] and our own previous work on this system [12] — and analyse the structure of initial-condition space. The system displays highly non-trivial multistable behaviour (as explored in other works [21, 22, 35]), in which three different dynamical attractors co-exist (Figure 3, a stroboscopic (Poincaré) plot of the different dynamics, sampled once a year, commensurate with the forcing period). A chaotic attractor exists extensively in initial-condition space and is interspersed with two different periodic attractors of period-3 and period-5 years [20].

We now examine which regions of initial condition space give rise to the alternative dynamical attractors. Figure 4 shows a scatterplot matrix of initial conditions *S*(0), *I*(0), *R*(0) and *W*(0) and the attractors to which they correspond. Red, green and blue dots respectively denote the chaotic, period-5 and period-3 attractors. The period-3 attractor maintains a similar structure throughout all possible combinations for the initial conditions. For example, our numerical sampling suggests that period-3 dynamics are confined to a limited and connected region of initial-condition space in the *S*-*I* cross-section, and show a compact, rectangular shape in different cross-sections (as in the *I*-*R* or *R*-*W* cross-sections). The other two attractors are instead widely dispersed without any coherent structure underpinning their morphology. These attractors appear riddled in initial-condition space, and in certain cross-sections also appear riddled with the period-3 orbits, although the degree of penetration of green and red points inside blue areas seems rather limited.The structure of the basins (Figure 4) has strong implications for the model dynamics and their instability to variations in population immunity, as can be appreciated by reconsidering the phase-space of these three attractors (Figure 3). This plot shows that, geometrically, the period-5 attractor (green) acts as a ‘support’ for the chaotic dynamics (red). The characteristic filament-like shape of chaos (red) appears to originate from and be woven around the period-5 orbit points (green dots). Note instead that the period-3 attractor (blue) is external and more distant from the other two. This picture, although it does not offer a rigorous justification for the shape of the basins shown in Figure 4, is in line with the more compact and less interspersed character of period-3 attractors. Given that period-5 and chaotic basins are shown to be intermingled in phase-space and strongly related geometrically, it is reasonable to expect that small variations in initial conditions may cause the dynamics to switch between the two attractors, whereas period-3 points will be relatively more ‘isolated’ in initial-condition space.

## Conclusions

From the point of view of possible strategies to prevent or control the spread of infectious diseases, our analysis is highly relevant. Consider, as Aron [22] has previously done, the common scenario of a ‘catch-up’ campaign in which a proportion of the susceptible population is vaccinated with a sterilizing vaccine (i.e. one which modifies, perhaps blocking entirely, susceptibility), in an impulsive fashion. Such a scenario is well modelled by shifting a proportion of the total population from the susceptible (*S*) to the recovered (*R*) state. From our perspective, this has the effect of instantaneously reducing *S*(0) and increasing *R*(0) while leaving *I*(0) and *W*(0) unchanged. Examination of Figure 4 suggests that whilst this approach might make sense intuitively as a control measure and would of course reduce the available pool of susceptible individuals at the time of program implementation, its effect over the longer term may be to move the observed dynamics from a regime where the period is 5 years to a scenario where the period is now 3 years and of much larger amplitude. This could potentially result in an increase in the burden of disease within the community, both in magnitude and frequency, due to the greater proportion of period-3 dynamics in that region of initial-condition space. Alternatively, a small perturbation in the initial conditions, perhaps due to stochastic noise, may lead to a scenario where the prevalence is fluctuating aperiodically, making the prediction (and control) of the disease burden increasingly difficult [21, 22]. The existence of dynamical crises suggests that it is possible that adverse effects may originate under the implementation of typical control strategies [21, 22]. It remains an open question as to whether any particular trends in observed infectious disease epidemiology (e.g. the observed resurgence of pertussis in Western nations over the past decade) may be (partially) attributed to such dynamical behaviour. Furthermore, our model does not include continual vaccination of birth cohorts. Modelling of standard childhood immunisation programs may impact on the structure of the basins.

Medium to long term experience of immunisation programs in diverse settings has confirmed that vaccine impact may change over time. Mathematical models have provided useful insights into the drivers of such observations, including the ‘honeymoon’ phenomenon [36] and shifts in herd protection following implementation of population catch-up programs [37]. Given the increasing use of models to inform policy decisions regarding vaccine introduction and scheduling and infectious diseases intervention policy more generally, our and others [21, 22, 38] findings highlight the importance of thoroughly investigating the dynamical properties of those models to identify key areas of uncertainty. These dynamical-systems considerations complement the well established finding (e.g., [39–41] that choices in model structure can strongly influence the estimated value(s) of model parameters and so interpretation of epidemiological data. Our findings suggest that the often stated tension between capturing biological complexity and utilising mathematically simple models is perhaps more nuanced than generally suggested. Simple dynamical models, particularly those which include forcing terms, can give rise to incredibly complex behaviour. But we must ask if that behaviour is ‘real’; is it observed or observable in the epidemiological data?

How to determine the appropriate level of mathematical detail for a model thus remains an open question. This is particularly pertinent at a time when the use of mathematical and computational models for the study of infectious diseases is rising as a major force in the development of public health policy. We suggest that at a minimum, gathering richer data on the epidemiological problem will aid in model selection and interpretation. Furthermore, post-implementation surveillance to monitor for ‘model-predicted’ dynamical changes to the system may be useful in order to refine model structures and parameterisations and either improve predictive capacity or highlight the inherent unpredictability of the system.
